# Reversible and Irreversible Cation Intercalation in NiFeO_*x*_ Oxygen Evolution Catalysts in Alkaline Media

**DOI:** 10.1021/acs.jpclett.2c03336

**Published:** 2023-01-11

**Authors:** Hanna Trzesniowski, Nipon Deka, Onno van der Heijden, Ronny Golnak, Jie Xiao, Marc T. M. Koper, Robert Seidel, Rik V. Mom

**Affiliations:** † Department of Atomic-Scale Dynamics in Light-Energy Conversion, Helmholtz-Zentrum Berlin für Materialien und Energie, 14109Berlin, Germany; ‡ Leiden Institute of Chemistry, Leiden University, P.O. Box 9502, 2300 RALeiden, The Netherlands; § Department of Highly Sensitive X-Ray Spectroscopy, Helmholtz-Zentrum Berlin für Materialien und Energie, 14109Berlin, Germany

## Abstract

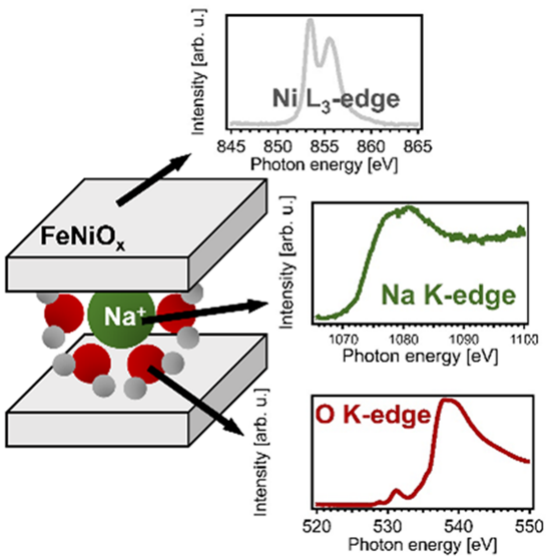

For electrocatalysts with a layered structure, ion intercalation is a common phenomenon. Gaining reliable information about the intercalation of ions from the electrolyte is indispensable for a better understanding of the catalytic performance of these electrocatalysts. Here, we take a holistic approach for following intercalation processes by studying the dynamics of the catalyst, water molecules, and ions during intercalation using operando soft X-ray absorption spectroscopy (XAS). Sodium and oxygen K-edge and nickel L-edge spectra were used to investigate the Na^+^ intercalation in a Ni_0.8_Fe_0.2_O_*x*_ electrocatalyst during the oxygen evolution reaction (OER) in NaOH (0.1 M). The Na K-edge spectra show an irreversible intensity increase upon initial potential cycling and a reversible intensity increase at the intercalation potential, 1.45 V_RHE_, coinciding with an increase in the Ni oxidation state. Simultaneously, the O K-edge spectra show that the Na^+^ intercalation does not significantly impact the hydration of the catalyst.

Layered materials are ubiquitous in nature and are widely used for energy conversion and storage.^1,2^ This class of materials is particularly attractive in the field of catalysis, since it provides a large accessible surface area.^3^ During an electrocatalytic reaction, redox processes may change the interlayer spacing related to the intercalation of electrolyte ions into the layers. Although catalytically inactive, the literature suggests that electrolyte ions may impede or enhance the rate of electrode reactions.^4−7^ For example, in Ni–Fe oxyhydroxide (NiFeO_*x*_H_*y*_), which exists as a layered double hydroxide, the catalytic activity increases with the cation size of the electrolyte (Li^+^ < Na^+^ < Cs^+^). Understanding the fundamental interactions of NiFeO_*x*_H_*y*_ with the electrolyte cations is important because this material is the best catalyst for oxygen evolution reaction (OER) in alkaline media due to its low overpotential and the involvement of earth-abundant elements.

The layered structure of Ni-based oxyhydroxide can be visualized from the Bode scheme (Figure 1).^8,9^ The scheme illustrates that the interlayer spacing varies with applied potential as the material gets oxidized. The oxidation of Ni(OH)_2_ to NiOOH is accompanied by uptake of ions from the electrolyte in order to maintain charge neutrality. Bode et al. suggested that Ba^2+^ and Na^+^ could be intercalated.^8^ Later, it was found that the γ-phase, where Ni species are present in the oxidation state 3+δ, is expanded by at least 0.34 nm compared to the β-phase lattice, which facilitates intercalation of cations.^10^ The intercalation of electrolyte ions into the lamellar structure of the catalyst has been confirmed through microgravimetric studies based on quartz crystal microbalance.^9,11,12^ Based on the mass variation with applied potential, it has been suggested that Li^+^ intercalates as a hydrated ion whereas K^+^ intercalates as a nonhydrated ion.^13^ The total mass increase was found to be independent of the nature of the cation. Another electrochemical quartz crystal microbalance (EQCM) study proposed that the cations do not participate in the initial oxidation of Ni(OH)_2_, but they intercalate only when the catalyst is cycled for a longer time, leading to highly spaced layers.^14^ Similar EQCM measurements produced contrasting results showing that the mass increase depends on the identity of the cation.^12^ In situ Ni L-edge and Fe L-edge XAS spectra have shown that the oxidation of Ni sites and OER onset is shifted to lower potentials by large cations like Cs^+^.^15^ This specific cation effect was in turn attributed to increase in electrolyte pH by larger cation due to the higher basicity of the corresponding alkali hydroxide, assuming that hydrated intercalated ions have a similar influence on the local pH. This recent hypothesis competes with other theories such as the stabilization of reactive intermediates by cations,^4,7^ blocking of active sites by hydrated cation clusters,^16^ alteration of interfacial water structure,^17^ and promotion of peroxo species by larger cations leading to a higher catalytic activity.^18^ Intercalated cations may provide access to intralayer active sites, as a variation on the theory of “blocking by hydrated cations”. Intralayer cations may have a different coordination environment compared to near-surface cations. This effect may lead to a change in the hydrogen-bonding network within and outside the catalyst thereby influencing its catalytic activity and redox behavior.

**Figure 1:**
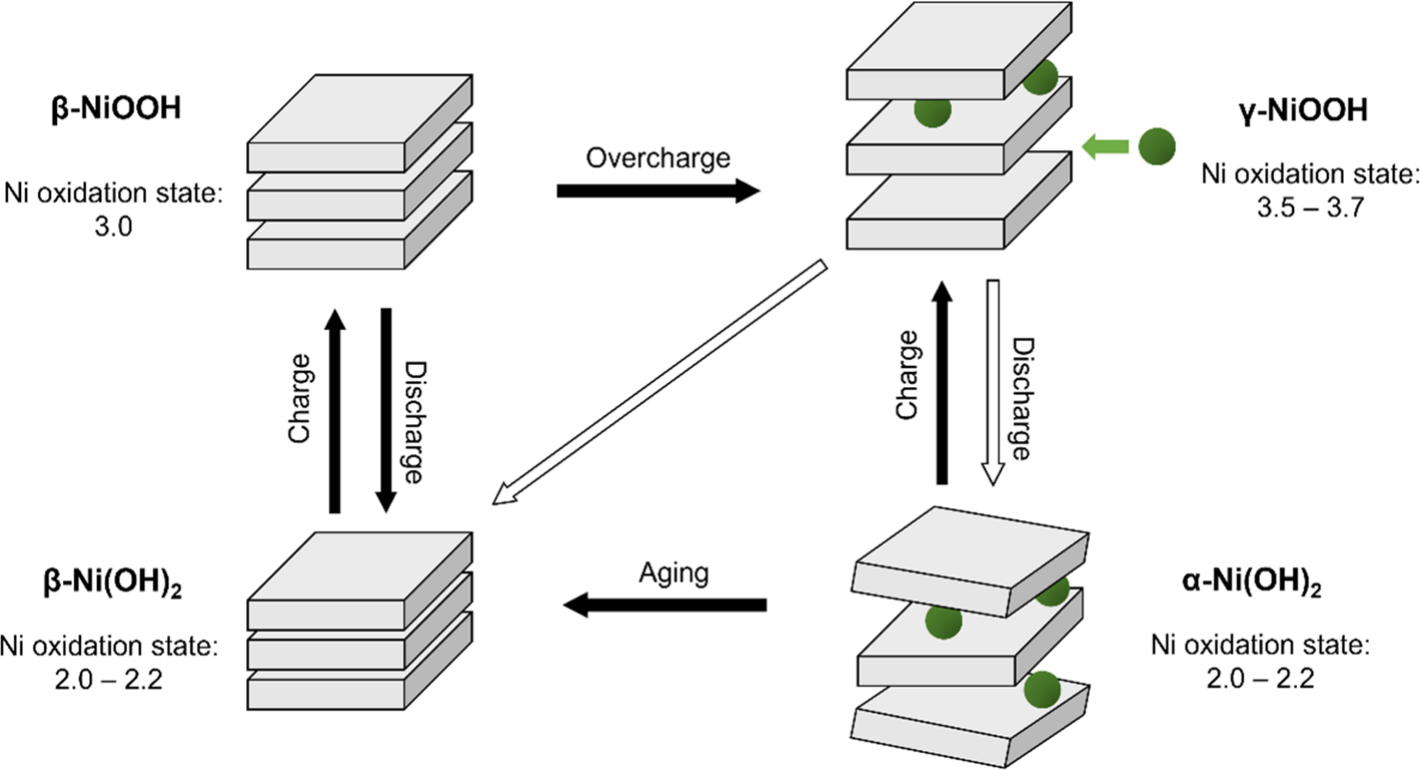
Four phases of Ni-based (oxy)hydroxide phases as proposed by Bode et al.^8^ Ions (herein represented in green) are able to enter the γ-NiOOH phase due to its increased interlayer space. Adapted with permission from ref (9).

Such discrepancies in interpretation may be the result of the lack of direct observation of cation intercalation. Mass changes during EQCM measurements may be caused by cations, anions, and/or water molecules and therefore microgravimetric measurements do not specifically probe a particular electrolyte constituent. To probe more specifically which species intercalate, we use in situ Na and O K-edge X-ray absorption spectroscopy (XAS) to directly probe the intercalation of Na^+^ cations and water into NiFeO_*x*_H_*y*_ at OER relevant potentials. The intensity of the Na K-edge spectrum can be directly related to the concentration of Na^+^ ions in the probed volume. The spectral shape also reveals information about the hydration shell of the Na^+^ cation. Simultaneously, O K-edge XAS probes the evolution of surface oxygen atoms in NiFeO_*x*_H_*y*_ and the influx of water molecules into the catalyst. Finally, in situ Ni L_2,3_-edge XAS probed the average oxidation state of the Ni sites. This approach thereby allows us to specifically probe the individual intercalating species and the change in catalyst structure caused by the influx of the cations at OER relevant potentials.

*Nickel Redox Behavior Probed via Cyclic Voltammetry and Nickel L-Edge XAS.* To obtain an understanding of the processes occurring at the electrocatalyst/electrolyte interface, we start by studying the redox chemistry of the Ni_0.8_Fe_0.2_O_*x*_ electrocatalyst by cyclic voltammetry (as shown in the inset in Figure 2). The cyclic voltammogram (CV) is reported between 1.0 and 1.7 V vs the reversible hydrogen electrode (RHE). The features at 1.3 and 1.4 V_RHE_ have been attributed to the Ni(OH)_2_/NiOOH redox couple.^9,19−22^ During this redox reaction, Ni^2+^ is converted to higher oxidation states. Around 1.5 V_RHE_, the current increases due to the onset of the oxygen evolution reaction. We note that no feature in the CV has been linked to iron. However, with increasing amount of iron in the electrocatalyst the redox waves have been found to decrease in area and to shift positively.^23−26^

**Figure 2:**
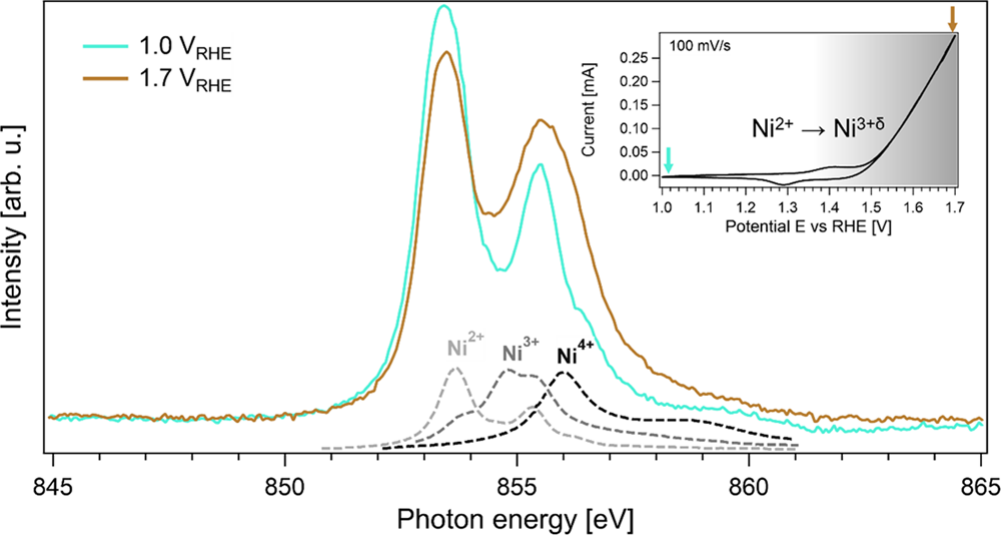
Operando Ni L_3_-edge spectrum recorded at 1.0 and 1.7 V_RHE_ (reference spectra taken from Qiao et al.^27^). Insert: Cyclic voltammogram of the electrocatalyst in 0.1 M NaOH recorded at 100 mV/s in the spectroelectrochemical cell.

To gain more insight into the potential-induced changes in the Ni oxidation state, we performed operando soft X-ray absorption spectroscopy at the Ni L_3_-edge. In Figure 2, we show spectra recorded at 1.0 V_RHE_ (below the Ni redox transition) and at 1.7 V_RHE_ (during OER catalysis). The Ni L_3_-edge spectrum recorded at 1.0 V_RHE_ shows the spectral features we expect for Ni^2+^ in an octahedral field: two dominant features at 853.3 and 855.3 eV and a shoulder above 856 eV. Thus, Ni is present in a +2 oxidation state at this potential in accordance with interpretation of the CV. Upon applying an OER potential of 1.7 V_RHE_, the resulting Ni L_3_-edge spectrum shows a peak weight shift to higher energies. This indicates an increase in the Ni valence state, which is also in agreement with the observations from the CV as well as expected from the literature on Fe-containing nickel (oxy)hydroxide catalysts.^28−35^ Comparing our measured spectrum at 1.7 V_RHE_ with calculated reference spectra by Qiao et al.^27^ provides evidence that we see mixed oxidation states of Ni^3+^ and Ni^4+^ at 1.7 V_RHE_. We note that only a part of Ni^2+^ is converted to higher oxidation states and that these oxidation states are not only +3 but +4 as well. This mixed oxidation state implies structural heterogeneity in the material and shows that the common Ni^2+^/Ni^3+^ assignment of the redox peaks in the CV is an oversimplification. Compared to the Bode diagram, the Ni L_3_-edge data suggest that a large part of the NiFeO_*x*_ film is in the γ-phase under OER conditions and thus should be able to incorporate Na^+^ ions.

*Sodium K-Edge XAS.* To follow the behavior of Na^+^ ions during the redox events in Ni_0.8_Fe_0.2_O_*x*_, we recorded Na K-edge X-ray absorption spectra (Figure 3). Figure 3a shows spectra recorded below (1.0 V_RHE_) and above (1.45 V_RHE_) the Ni redox transition. The spectrum recorded at 1.45 V_RHE_ shows an increased intensity in regions B and C compared to the spectrum recorded at 1.0 V_RHE_ revealing a higher concentration of Na^+^ in the probing depth. This jump in intensity confirms that sodium ions enter the catalyst layer at potentials above the Ni redox transition. When a potential of 1.0 V_RHE_ is applied again (darker green spectrum in Figure 3a), the intensities decrease to the initial values. Hence, we conclude that this intercalation occurring during Ni redox is fully reversible.

**Figure 3:**
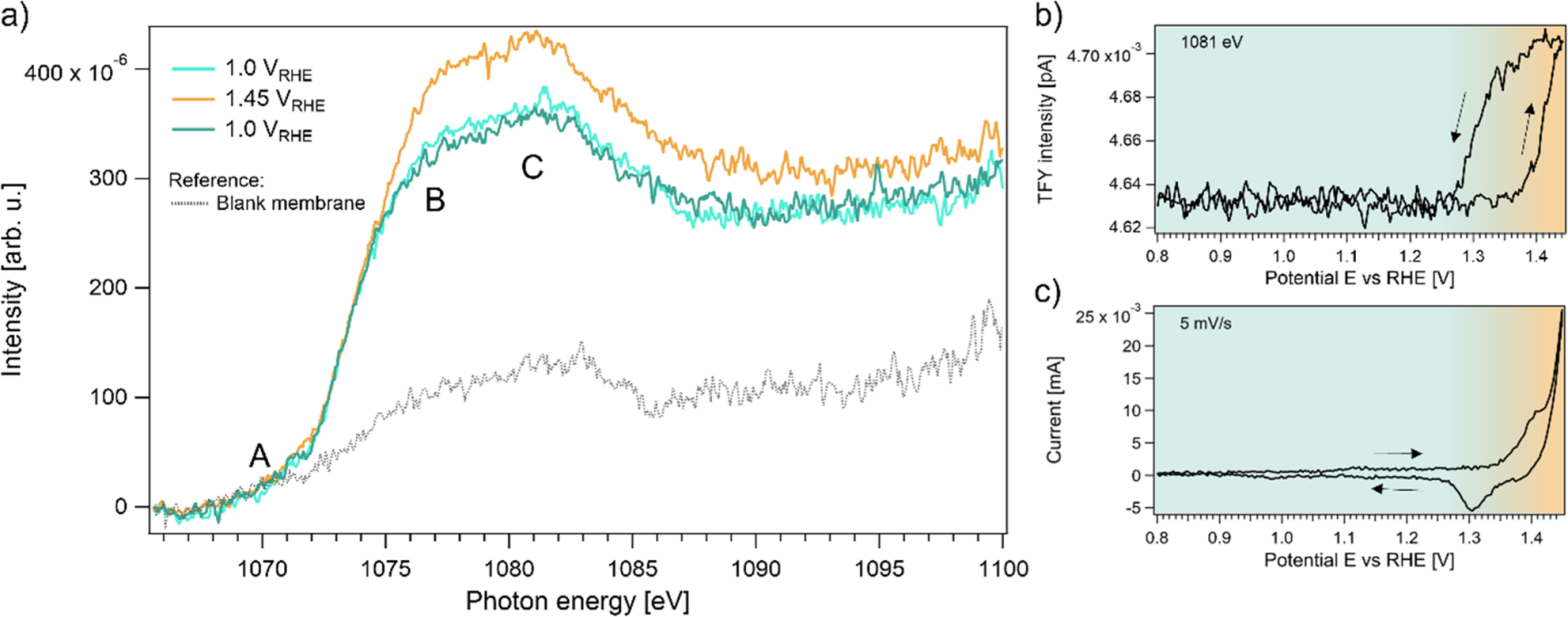
(a) Operando sodium K-edge spectra of the electrocatalyst in 0.1 M NaOH recorded at 1.0, 1.45, and again at 1.0 V_RHE_ and Na K-edge spectrum of the blank membrane in NaOH for comparison, (b) TFY intensity of the spectral feature C at 1081 eV as a function of potential between 0 and 1.45 V_RHE_. (c) Corresponding cyclic voltammogram recorded at 5 mV/s (arrows indicate the scan direction)

By comparing the Na K-edge spectrum recorded at 1.0 V_RHE_ with that of a blank membrane (i.e., not coated with NiFeO_*x*_) in 0.1 M NaOH, it is clear that even at low potentials there is a significant amount of Na^+^ in the Ni_0.8_Fe_0.2_O_*x*_ film. Otherwise, the observed Na^+^ signal within the volume probed by XAS could not be higher. From the spectra in Figure 3 it is evident that the difference is large. The real difference in concentration is even higher than indicated by this figure, because a smaller electrolyte volume is probed when the membrane is coated with NiFeO_*x*_ due to the volume that the NiFeO_*x*_ occupies and due to the lower probing depth when NiFeO_*x*_ is present. Thus, contact with the electrolyte and/or initial potential cycling leads to irreversible Na^+^ intercalation. In combination with the Ni^2+^ oxidation state observed in the Ni L-edge, this observation suggests that the Ni_0.8_Fe_0.2_O_*x*_ film at 1.0 V_RHE_ has a structure analogous to the α-Ni(OH)_2_ phase in the Bode model in Figure 1. Along the same lines, the Ni^2+/3+/4+^ state and the high Na^+^ content at 1.45 V_RHE_ suggest a structure similar to the γ-NiOOH phase in Figure 1.

The presence of the Na^+^ ions in the structure indicates that the Ni oxide is negatively charged. In alkaline media, this is readily rationalized: part of the OH groups in the material will be deprotonated by OH^–^ ions from the electrolyte:
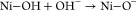
1Following this acid–base reaction, the Na^+^ enters the structure to maintain overall charge neutrality. Interestingly, the observation of an increased number of Na^+^ ions in the γ-NiOOH-like phase at 1.45 V_RHE_ indicates that it carries a higher negative charge than the α-Ni(OH)_2_-like phase, even though it has fewer OH groups. We hypothesize that the applied potential reduces charge density at the OH groups resulting in increased acidity which in turn favors the formation of Ni–O^–^. Indeed, in situ Raman spectroscopy on Ni oxide suggested the presence of OO^–^ species in the γ-NiOOH phase.^36^ By studying the shape of the Na K-edge spectra, further information on the hydration shell of the intercalated ions can be obtained.

The ratio of peak intensity C to peak intensity B in the Na K-edge strongly depends on the number of coordinating water molecules around Na^+^.^37^ In Figure 3, the B:C ratio remains almost constant with a value of 1.06 at 1.0 V_RHE_ and 1.09 at 1.45 V_RHE_. This indicates that the number of water molecules in the first solvation shell is the same at both potentials. Hence, the intercalation structure of the ions appears to remain constant during the α/γ phase transition; only the number of intercalated ions changes.

To gain potential-resolved insight into the intercalation behavior, we tracked the TFY intensity of feature C while cycling the electrode potential between 0 and 1.45 V_RHE_ at a scan rate of 5 mV/s (Figure 3b). By comparing the TFY intensity as a function of potential with the corresponding CV (Figure 3c), it is evident that the Na^+^ intercalation and deintercalation and the Ni redox process are directly correlated (for further analysis see Supporting Information section SI 2). This indicates that the formation of additional negatively charged groups (either O^–^ or OO^–^) that drives Na^+^ intercalation proceeds one-to-one with the overall redox process. Furthermore, our data confirm that no Na^+^ intercalation occurs in other potential regions, consistent with the lack of features in the cyclic voltammogram.

*Oxygen K-Edge XAS.* To monitor changes related to oxygen-containing species in the electrocatalytic system during the intercalation process, we recorded spectra at the O K-edge. The O K-edge X-ray absorption spectrum in Figure 4 shows five distinct features, labeled A–E. The spectral features A and B correspond to the transition metal oxide catalyst, while the regions C, D, and E are attributed mainly to the aqueous electrolyte.

**Figure 4:**
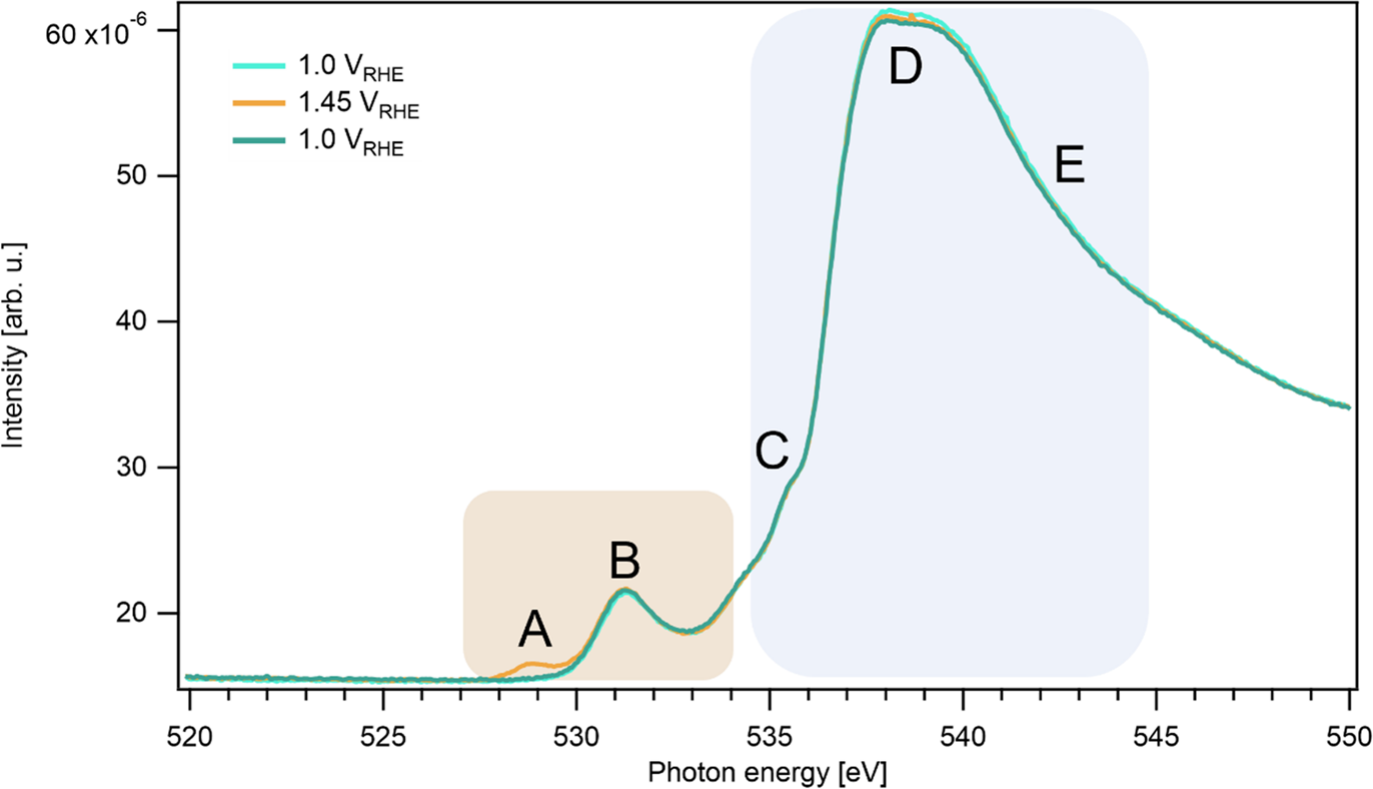
Operando oxygen K-edge spectra of Ni_0.8_Fe_0.2_O_*x*_ recorded at 1.0, 1.45, and again at 1.0 V_RHE_. Features in the brown shaded area are associated with the oxide catalyst, and features in the blue shaded area are dominated by water.

The oxide features A and B are highly sensitive to the type of bonds the oxygen forms with the 3d transition metals. Feature B has been found in all 3d transition metal (TM) oxides and is assigned to the excitation from the O 1s core orbital to the hybridized O 2p–TM 3d state.

At an increased electrode potential of 1.45 V_RHE_ an additional feature appears in region A, which disappears reversibly upon application of a potential of 1.0 V_RHE_. This feature has been linked to the presence of electron-deficient oxygen sites.^38^ Such sites are likely important for OER catalysis, as electron-deficient O or OH groups are required for the O–O coupling step in the reaction. Similar to the Na^+^ intercalation, the formation of the electron-deficient oxygen appears to be correlated to the Ni redox.

The intensity of the spectral features in the water region (blue shaded area) does not significantly change as a function of applied potential. A minute decrease in region D due to oxygen bubble formation is visible over time, but it is uncorrelated to the potential.

The observation that the applied potential has no effect on the intensity and shape of the water-region leads to the conclusion that the total amount of water inside the catalyst structure is constant. Hence, the intercalation of Na^+^ ions does not appear to lead to additional spacing between the nickel oxide layers that could accommodate extra water molecules. We hypothesize that the structure is already rather open at potentials below the Ni redox peak. This would be in line with the observation that the structure already contains a significant amount of Na^+^ ions at 1.0 V_RHE_.

We used operando soft X-ray absorption spectroscopy to observe the different processes occurring at the electrocatalytic interface of a NiFeO_*x*_ catalyst film in 0.1 M NaOH at potentials relevant for OER. By recording Ni L-edge, Na K-edge, and O K-edge spectra, we were able to investigate all components of the electrode–electrolyte interface individually. Ni L-edge XAS showed that Ni^2+^ dominates at 1.0 V_RHE_, whereas a mixture of Ni^2+/3+/4+^ is formed at 1.7 V_RHE_. The Na^+^ content in the structure increased irreversibly upon initial potential cycling and increased reversibly following the Ni oxidation. The reversible increase in intercalated Na^+^ did not lead to an increase in the water content of the catalyst layer, suggesting that the Ni redox and accompanying Na^+^ intercalation have little effect on the catalyst morphology.

Overall, our XAS data support electrochemical studies suggesting that Na ions from the electrolyte are being intercalated into the γ-phase of Ni-based electrocatalysts for water oxidation.

## Experimental Methods

*Sample Preparation.* The NiFeO_*x*_ catalyst was dip coated on a silicon nitride window according to the following procedure. First, 0.005 mmol of PEO_104_-PB_92_-PEO_104_ (PSM03) was dissolved in ethanol and stirred for 30 min at 45 °C. Then, 0.75 mmol of citric acid (Sigma-Aldrich), 1.16 mmol of Ni(NO_3_)_2_·6 H_2_O (Sigma-Aldrich), and 0.29 mmol of Fe(NO_3_)_3_·9 H_2_O (Sigma-Aldrich) were added at the same time and stirred for 1.5 h at room temperature. 75 nm thin SiN_*x*_ membranes (0.5 mm × 0.5 mm, Silson Ltd.) supported by a Si frame (10 mm × 10 mm × 381 μm) and covered with a 5 nm Ti adhesion layer and 20 nm Au conductive layer were used as substrates. These substrates were coated with the solution via dip coating, which was performed on a home-built device under Ar atmosphere in a glovebox to exclude humidity. To ensure that only the electrolyte-facing side is coated with sample, the SiN_*x*_ membranes were mounted onto a silicon wafer by means of Kapton tape. Thus, a frame of tape surrounded each membrane when they were submerged into the dip coating solution. After drying for 10 min, the samples were transferred in a closed vessel under Ar atmosphere from the glovebox to a preheated oven, where they were treated at 250 °C for 1 h. Subsequently the samples were calcined at 350 °C for 1 h under air flow in a preheated muffle furnace converting the precursor into nickel iron oxide. After the calcination step, the Kapton tape was gently removed. By controlling the withdrawal rate of the substrates from the dip coating solution, the substrates were coated with an amount of precursor solution that resulted in a thickness of the calcined film of about 100 nm. Details on determination of the film thickness are given in the Supporting Information section SI 3. Ex situ XAS spectra of the as-synthesized sample can be found in the Supporting Information section SI 4.

**Figure 5:**
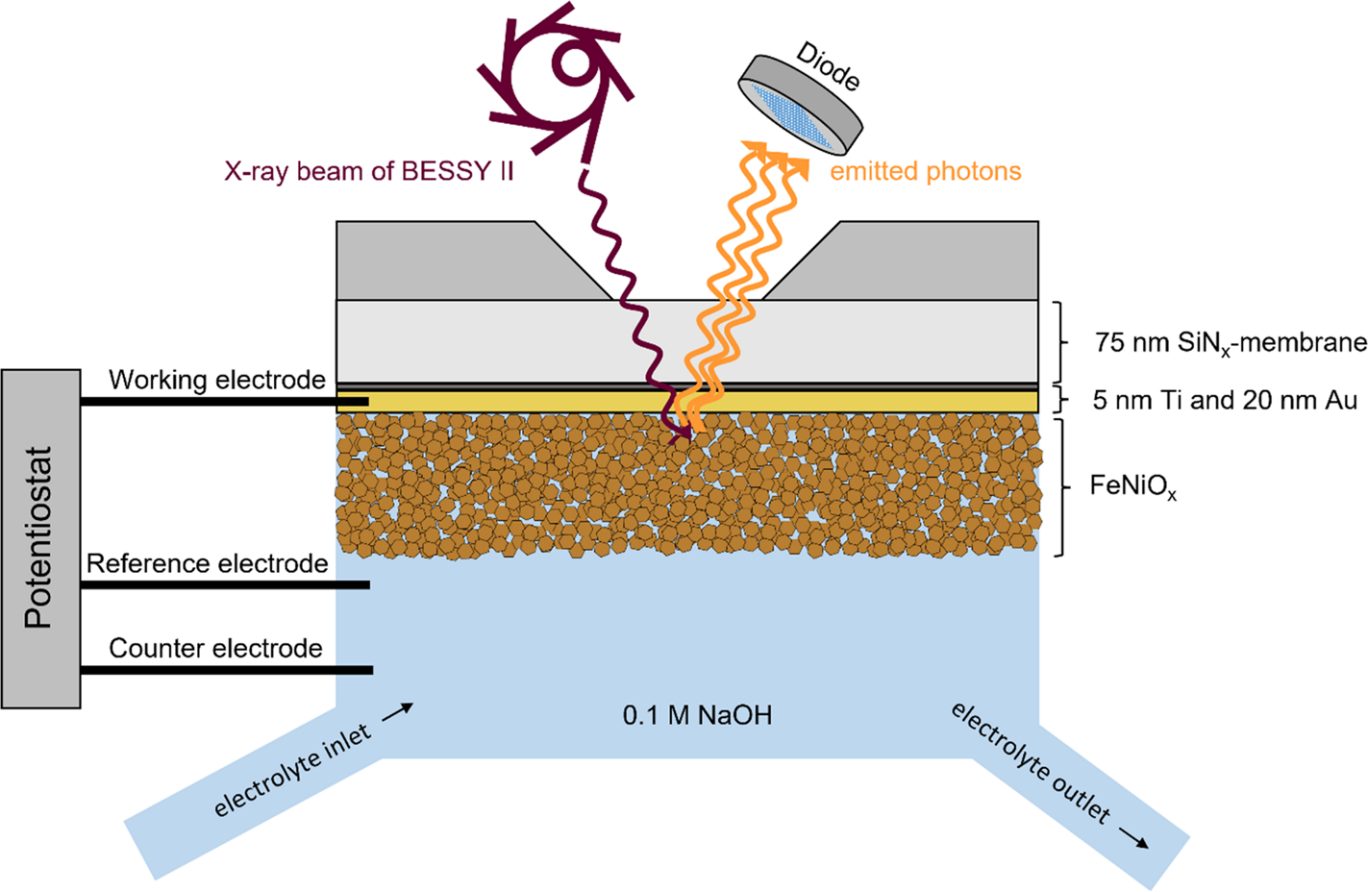
Schematic illustration of the electrochemical flow cell. The three-electrode setup enables applying potentials while spectra are recorded through the photon-transparent SiN_*x*_ membrane. The SiN_*x*_ membrane, which is coated with Ti and Au, acts simultaneously as the working electrode and as the substrate for the FeNiO_*x*_ layer.

*Operando X-ray Spectroscopy.* The operando O K-edge XAS, Ni L-edge XAS, and Na K-edge XAS were recorded in total fluorescence yield (TFY) mode with the LiXEdrom setup^39^ at the soft X-ray beamline U49-2_PGM-1 at the synchrotron-radiation facility BESSY II, Berlin, Germany. All operando X-ray absorption spectra were recorded in an electrochemical flow cell designed by Tesch et al.^40^ A scheme of the setup is displayed in Figure 5. SiN_*x*_ membranes coated with the iron nickel oxide electrocatalyst separate the electrolyte from the surrounding vacuum. The Au-layer on top of the SiN_*x*_ membranes serves as the connection for the working electrode, and a leak-free Ag/AgCl electrode (Innovative Instruments, Inc. LF-1.6, 3.4 M AgCl) was used as the reference electrode and a Pt-wire as counter electrode. Potentials were applied with a BioLogic potentiostat (SP-200, BioLogic Scientific Intruments). The electrolyte, 0.1 M Fe-free NaOH (previously cleaned following the procedure described by Trotochaud et at.,^25^ starting electrolyte: 30 wt % NaOH in water, Suprapur), was pumped through the cell via a syringe pump at a rate of 50 μL/min. The resistance in the flow cell was approximately 350 Ω. The samples were treated by cycling 100 times between 1 and 1.7 V_RHE_ at 100 mV/s before recording any XAS spectra. All spectra were normalized to the incoming photon flux.

## Supporting Information Available

Additional Na K-edge spectra of the Ni_0.8_Fe_0.2_O_*x*_ film in water and air, further analysis of the Na signal at 1081 eV, information about the determination of film thickness, and ex situ spectra at the Ni L-edge and O K-edge of the as-synthesized sample (PDF)
