# Supplementary material for: Reversible and Irreversible Cation Intercalation in NiFeOx Oxygen Evolution Catalysts in Alkaline Media
Source: J Phys Chem Lett. 2023 Jan 11;14(2):545–51. doi: 10.1021/acs.jpclett.2c03336 (PMC9870210; doi:10.1021/acs.jpclett.2c03336)
Supplement: Supplementary file 1 — jz2c03336_si_001.pdf [file jz2c03336_si_001.pdf]

Supporting Information to

The Reversible and Irreversible Cation Intercalation in $NiFeO_x$ Oxygen Evolution Catalysts in Alkaline Media

Hanna Trzesniowski[1], Nipon Deka[2], Onno van der Heijden[2], Ronny Golnak[3], Jie Xiao[3], Marc T.M. Koper[2], Robert Seidel[1], Rik V. Mom[2]**

1. Department of Atomic-Scale Dynamics in Light-Energy Conversion, Helmholtz-Zentrum Berlin für Materialien und Energie, 14109, Berlin, Germany.
2. Leiden Institute of Chemistry, Leiden University, PO Box 9502, 2300 RA Leiden, The Netherlands.
3. Department of Highly Sensitive X-Ray Spectroscopy, Helmholtz-Zentrum Berlin für Materialien und Energie, 14109, Berlin, Germany.

Contents

SI 1 Na K-edge spectra in water and air S2
SI 2 Analysis of Na signal at 1081 eV S3
SI 3 Determination of film thickness S4
SI 4 Ex-situ Ni L-edge and O K-edge spectra of the as-synthesized sample S5

SI 1 Na K-edge spectra in water and air

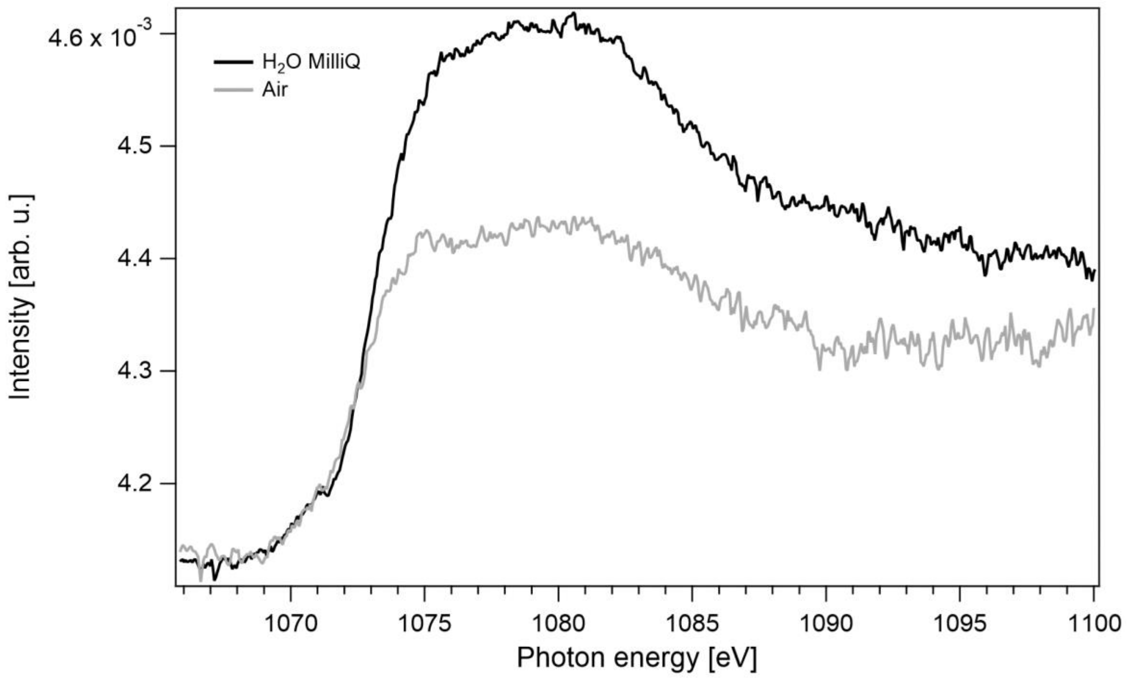

Fig. S1. Sodium K-edge spectra of the $NiFeO_x$ electrocatalyst in MilliQ water and in air (i.e. in the empty flow-cell) after performing operando XAS measurements in 0.1 M NaOH.

To study the reversibility of the Na intercalation, we exchanged NaOH for MilliQ water after recording operando XAS spectra. NaOH was replaced by changing the syringe to a water-filled syringe and flushing the flow-cell with an amount that equals three times the volume of the flow-cell. After the spectra in water were recorded, we changed the syringe to an empty syringe and sucked out the liquid through reverse pumping. From these spectra it is clear that the Na intercalation is not fully reversible and that a certain amount of sodium ions stay in the structure even after the electrolyte is removed.

SI 2 Analysis of Na signal at 1081 eV

For the sake of clarity, the TFY signal at 1081 eV as a function of potential and the corresponding CV (0 - 1.45 V vs RHE, 5 mV/s) are directly compared. From this representation it is evident that the oxidation process of Ni is directly correlated to the intercalation of Na (purple line) and the reduction is correlated to the deintercalation (green line).

The derivative of the TFY signal is shown in Fig SI2b. From this comparison it is clear that the current signal and the derivative of the TFY signal coincide. The peak of the oxidation wave in the CV and the local maximum of the derivative occur at the same potential as well as the peak of the reduction wave and the local minimum of the TFY signal.

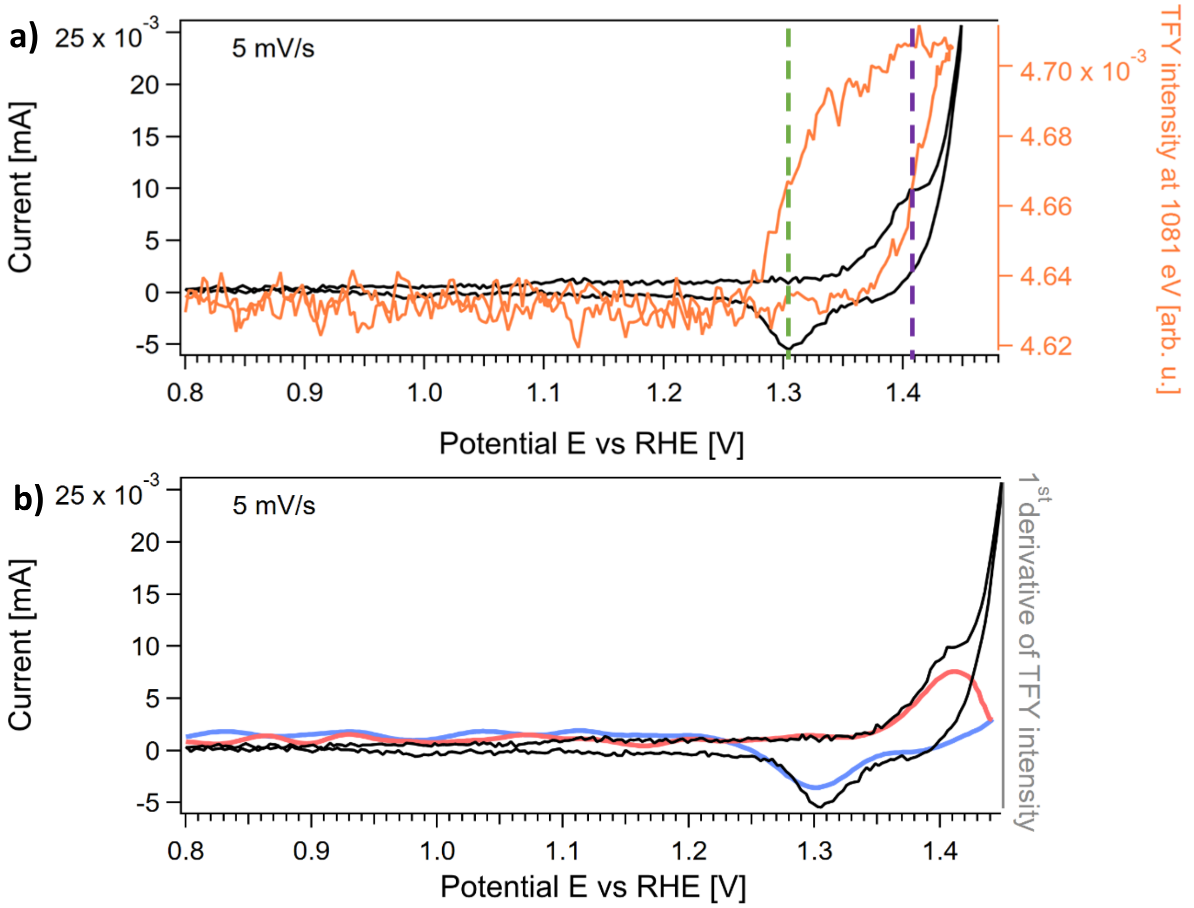

Fig. S2. a) Direct comparison of TFY intensity at 1081 eV as a function of potential and the corresponding CV. Dashed vertical lines mark the oxidation/intercalation (purple) and the reduction/deintercalation (green). b) First derivative of TFY intensity at 1081 eV (graph was smoothened for better visibility of the peaks) compared with the corresponding CV.

SI 3 Determination of film thickness

A silicon wafer was dip coated with the same precursor solution and the same withdrawal rate as the substrates for operando XAS. After being exposed to the same thermal treatment, the wafer was broken in the middle and the cross section was examined with a scanning electron microscope (JEOL 7401F, accelerating voltage: 10 kV).

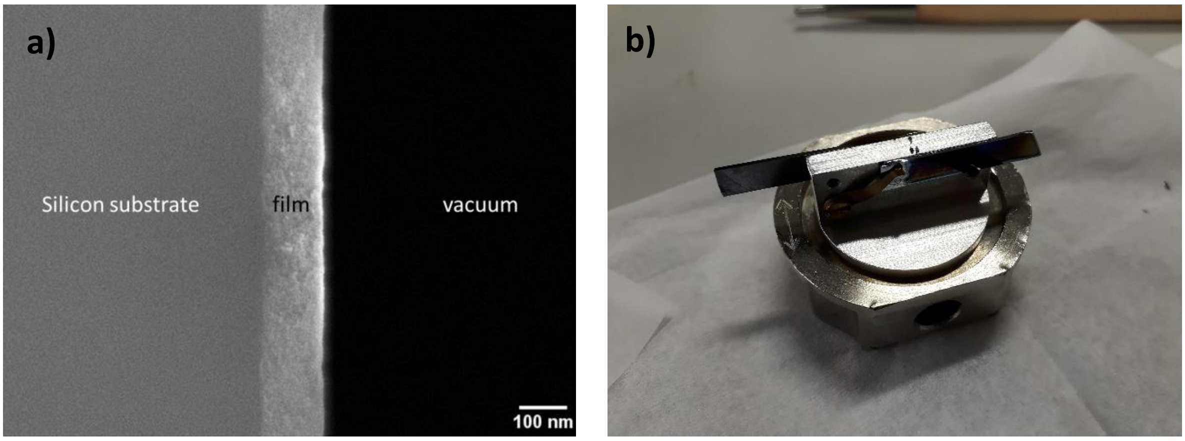

Fig. S3. a) Electron micrograph of the cross section of the $Ni_{0.8}Fe_{0.2}O_x$ film on a silicon wafer. b) The SEM sample holder with a sample for film thickness determination.

SI 4 Ex-situ Ni L-edge and O K-edge spectra of the as-synthesized sample

Ex-situ spectra of the as-synthesized sample were recorded on a sample holder, i.e. not in the electrochemical flow cell.

The Ni L-edge of the pristine sample exhibits four prominent absorption features at 853.3, 855.1, 870.3 and 871.6 eV corresponding to Ni in the +2 oxidation state. In the Ni L-edge spectrum, the main difference between the pristine (Fig. S4a) and the cycled sample (Fig. 2 in the main text) is the intensity ratio of the two dominant features in the L_3-region. The shoulder around 855 eV rises in intensity after the sample has been electrochemically treated. The enhancement of this shoulder can be explained by the presence of hydroxide species on the sample surface and/or the presence of a small fraction of Ni^{3+} species.

The O K-edge of the pristine sample shows a characteristic feature at 531.8 eV and a broad spectral feature from 535 eV onwards. In the O K-edge spectrum of the cycled catalyst in contact with KOH (0.1 M), there is significant increase in intensity in the region around 540 eV compared to the feature around 532 eV. Thus, the main contribution around 540 eV is attributed to the presence of the aqueous electrolyte.

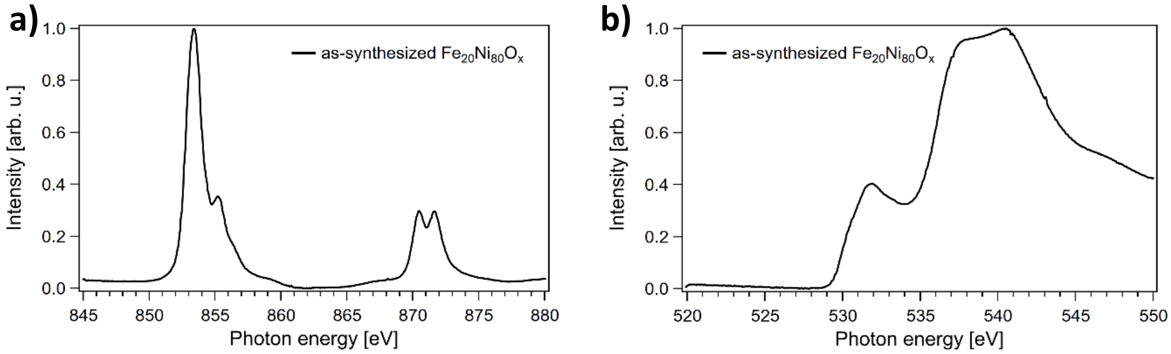

Fig. S4. a) Ex-situ Ni L-edge spectrum of the as-synthesized (i.e. uncycled) catalyst in vacuum, recorded in transmission mode. b) Ex-situ O K-edge spectrum of the as-synthesized (i.e. uncycled) catalyst in vacuum, recorded in transmission mode.
